# p53 Selectively Regulates Developmental Apoptosis of Rod Photoreceptors

**DOI:** 10.1371/journal.pone.0067381

**Published:** 2013-06-20

**Authors:** Linda Vuong, Daniel E. Brobst, Ivana Ivanovic, David M. Sherry, Muayyad R. Al-Ubaidi

**Affiliations:** 1 Department of Cell Biology, University of Oklahoma Health Sciences Center, Oklahoma City, Oklahoma, United States of America; 2 Oklahoma Center for Neurosciences, University of Oklahoma Health Sciences Center, Oklahoma City, Oklahoma, United States of America; 3 Department of Pharmaceutical Sciences, University of Oklahoma Health Sciences Center, Oklahoma City, Oklahoma, United States of America; University of Florida, United States of America

## Abstract

Retinal cells become post-mitotic early during post-natal development. It is likely that p53, a well-known cell cycle regulator, is involved in regulating the genesis, differentiation and death of retinal cells. Furthermore, retinal cells are under constant oxidative stress that can result in DNA damage, due to the extremely high level of metabolic activity associated with phototransduction. If not repaired, this damage may result in p53-dependent cell death and ensuing vision loss. In this study, the role of p53 during retinal development and in the post-mitotic retina is investigated. A previously described super p53 transgenic mouse that expresses an extra copy of the mouse p53 gene driven by its endogenous promoter is utilized. Another transgenic mouse (HIP) that expresses the p53 gene in rod and cone photoreceptors driven by the human interphotoreceptor retinoid binding protein promoter was generated. The electroretinogram (ERG) of the super p53 mouse exhibited reduced rod-driven scotopic a and b wave and cone-driven photopic b wave responses. This deficit resulted from a reduced number of rod photoreceptors and inner nuclear layer cells. However, the reduced photopic signal arose only from lost inner retinal neurons, as cone numbers did not change. Furthermore, cell loss was non-progressive and resulted from increased apoptosis during retinal developmental as determined by TUNEL staining. In contrast, the continuous and specific expression of p53 in rod and cone photoreceptors in the mature retinas of HIP mice led to the selective loss of both rods and cones. These findings strongly support a role for p53 in regulating developmental apoptosis in the retina and suggest a potential role, either direct or indirect, for p53 in the degenerative photoreceptor loss associated with human blinding disorders.

## Introduction

p53 is a tumor suppressor that is activated in response to cellular stressors such as DNA damage, oncogene activation, and loss of contact between cells (for review [1]). Its main functions include cell cycle arrest in response to cell stress and facilitating the repair of damaged DNA. If the damage cannot be repaired, p53 initiates apoptosis through mitochondrial membrane permeabilization and the caspase cascade [2].

Although p53 is known to be expressed in different ocular tissues [3], [4], the absence of p53 in C57BL×CBA [5] and 129/Sv×C57BL/6 [6] mice does not lead to any ocular abnormalities, implying either that other p53 family members compensate for its absence or that p53 may not be essential for eye development. However, severe ocular abnormalities arise in the p53 null mouse in the C57BL/6 and BALB/c OlaHsd backgrounds, suggesting that alleles from the C57BL/6 genetic background contribute to the observed phenotypes in the absence of p53 [7]. This implies that p53, or the pathway in which it functions, is important for normal development and/or maintenance of the eye [7].

During early embryogenesis in the mouse, p53 is expressed at high levels but as cells exit the cell cycle and terminally differentiate, p53 transcript and protein levels decline [8]. Similarly, the steady state levels of p53 in the developing mouse eye are highest at embryonic days (E) 17 and 18, drop precipitously to very low levels and then remain at those low levels throughout adulthood [9]. Although this finding suggests a role for p53 in early retinal development, it is not clear what role p53 plays beyond E18, the peak of differentiation of retinal cells [10], during postnatal retinal development, or in the mature retina. Furthermore, p53 may have important roles in the retina during stress or disease although these potential roles remain unclear. Although p53 may be dispensable for light- or chemical stress-induced apoptosis and in certain animal models of retinitis pigmentosa (RP), p53 has been linked to retinal responses to irradiation, oxidative stress, and the development of retinoblastoma ([11]for review).

To better understand the role of p53 in the developing retina and the significance of its downregulation in post-mitotic retinal cells, we studied the developing and adult retina in the super p53 mouse, a p53-overexpressing transgenic mouse model that has been previously characterized [12], and in a newly generated transgenic mouse model that overexpresses p53 specifically in retinal photoreceptors from mid-embryonic stages into adulthood (HIP, **H**uman **I**nterphotoreceptor retinoid binding protein promoter-**P**53).

We demonstrate that the super p53 mouse exhibits increased developmental retinal apoptosis, supporting an important role for p53 in retinal development. This p53-induced developmental apoptosis decreased the total number of rod photoreceptors and postreceptoral neurons in the inner nuclear layer (INL), but did not induce loss of cone photoreceptors or any further progressive degeneration of retinal cells in the mature retina. These changes were also reflected in the functional responses recorded from the super p53 mouse. To specifically assess the effects of p53 expression in adult photoreceptors, and to test whether the site of transgene integration was important to p53 overexpression effects, retinal structure and function also were examined in HIP transgenic mice, and corroborated the findings from the super p53 mouse.

## Materials and Methods

### Animals

All animal experiments were approved by the University of Oklahoma Health Sciences Center Institutional Animal Care and Use Committee (IACUC) and conformed to the National Institute of Health Guide for the Care and Use of Laboratory Animals and the Association for Research in Vision and Ophthalmology Resolution on the Use of Animals in Research. Animals were maintained at 30 to 50 lux on a 12-hour day/light cycle unless otherwise specified. Food and water were available ad libitum. Details for each mouse line are provided below.

### Super p53 Mice

Super p53 transgenic mice were a gracious gift from Dr. Manuel Serrano (Spanish National Center of Biotechnology, Madrid, Spain) [12]. These transgenic mice were created using a 130 kb segment of genomic p53 that is under endogenous control regardless of the site of integration [13]. In functionality tests, the transgene was able to rescue p53 deficiency in mouse embryonic fibroblasts and in p53 null mice [12]. Super p53 mice showed enhanced responses to DNA damage and were more tumor resistant than wild-type controls but maintain normal aging processes [12].

### Transgenic HIP Mice

A high-fidelity PCR cloning strategy was used to assemble a construct in which the human interphotoreceptor binding protein (hIRBP) promoter drives the expression of *Trp53*, the murine p53 gene, specifically in rod and cone photoreceptors of the retina from E10 onward. Briefly, a 1.4 kb hIRBP promoter was amplified from the plasmid using a primer set in which the forward primer (5′-CCACGTCCCTGAGACCACCTTCTCCAGTCGACGCTGCCTACTGAGGCACACA-3′) corresponds to the hIRBP sequence and the reverse primer (5′-GAGAGAAGAGATTGTGTACTGTATGGATGCTGGTGGACAGAAGGTCTGGGGCTAAAC-3′) was chimeric with the hIRBP sequence followed by an overhang encompassing the 5′-end of the *Trp53* sequence. Likewise, a 5.1 kb fragment encompassing exons 2–11 of the *Trp53* gene [14] was amplified using a forward primer (5′-GTTTAGCCCCAGACCTTCTGTCCACCAGCATCCATACAGTACACAATCTCTTCTCTC-3′) that was complementary to the reverse primer used above to amplify the hIRBP sequence. The corresponding reverse primer (5′-TCTCAAAGAGGCTTAGTCGACTGACTCCAACAGACTGCCTGGAC-3′) was complementary to the 3′-end of the *Trp53* sequence. The transgenic vector was sequenced to ensure the absence of any mutations prior to injection into mouse embryos. The general strategy for the generation of the construct and the transgenic mice was previously published [15].

Potential transgenic founders (F_0_) and their offspring (F_1_ and later) were identified by PCR screening. Briefly, DNA was extracted from ear punches and used as the template for an amplification reaction in the presence of an hIRBP-specific primer (5′-AGACCTTCTGTCCACCAGCA-3′) and a transgene-specific primer (5′-GATCGTCCATGCAGTGAGGTG-3′). To ensure the transgene was passed through the germ line, potential founder mice were mated to in-house inbred normal (wild-type) mice [16].

Southern blot analysis was used to determine both the pattern of transgene integration and copy number in the transgenic lines. Comparative quantitative densitometry measurements of the transgene and endogenous specific bands on the blots were used to determine the transgene copy numbers.

All mice were either in the C57BL/6 background or in our in-house inbred breeder strain [16].

Due to a malfunction in the HVAC system of the mouse facility, the ambient temperature increased leading to the accidental death of many mouse lines including all the HIP mice.

### The Retinal Degeneration (rd/rd) Mouse

Stocks of the *rd/rd* mouse were obtained from Jackson Laboratories (Bar Harbor, ME) and raised under conditions as outlined above. These mice were crossed to the HIP mice and then backcrossed to establish *rd*/*rd* mice that express HIP.

### Immunoblot Analysis

Sample preparation and immunoblotting were performed as previously described [15]. Briefly, primary antibodies were diluted in blocking solution and blots were incubated overnight at 4°C or 1 hour at room temperature. Appropriate secondary antibodies were applied for 1 hour at room temperature, and detection was performed using an ECL-detection kit according to the manufacturer’s instructions (Pierce, Rockford, IL) and a Kodak Image Station 4000R (Eastman Kodak, Rochester, NY). Details of the antibodies used for immunoblot analysis are provided in Table 1.

**Table 1:** Antibodies used for immunoblot analysis.

Antibody	Dilution	Source	Catalog Number
Monoclonal p53	1∶1000	Cell Signaling Technology	2524
Polyclonal p53	1∶2000	Santa Cruz	sc-6243
p63	1∶1000	Santa Cruz	sc-8343
p73	1∶1000	Santa Cruz	sc-7957
Mdm2	1∶1000	EMD Biosciences	OP46T
Mdm4 [48]	1∶2000	Dr. Steven J. Berberich, Boonshoft School of Medicine, Wright State University, Dayton, OH	
Actin	1∶1000	Abcam	ab6276
Anti-rabbit	1∶10000	KPL	0751–1506
Anti-mouse	1∶10000	Amersham ECL	NA931

### Light and Electron Microscopy

Eyes were collected, processed for Spurr’s resin embedment and microtomy, and examined by light and electron microscopy as previously described [17]. For light microscopy, 0.75 µm to 1 µm thick tissue sections were stained with toluidine blue and examined using an Olympus BH-2 photomicroscope (Olympus America, Center Valley, PA) and photographed using a Nikon DXM-1200 digital camera system (Nikon, Inc., Tokyo, Japan). For electron microscopy, silver-gold tissue sections were examined with a JEOL 100 EX transmission electron microscope (JEOL, Peabody, MA).

### Immunohistochemistry and Lectin Cytochemistry

Samples for immunohistochemistry (IHC) and lectin labeling were processed as previously described [18]. Details of the antibodies used for fluorescent immunolabeling and lectins are provided in Table 2.

**Table 2:** Antibodies and lectins used for fluorescence IHC labeling.

Antibody	Host	Dilution	Source	Catalog Number
Monoclonal p53	mouse	1∶10	Oncogene Research Products	OP29
Monoclonal p53	mouse	1∶2000	Cell Signaling Technology	2524
Polyclonal p53	rabbit	1∶50–1∶500	Santa Cruz	sc-6243
Mdm2	mouse	1∶20–1∶100	Oncogene Research Products	OP46 IF2
Mdm4 [48]	rabbit		Dr. Steven J. Berberich, Boonshoft School of Medicine,Wright State University, Dayton, OH	
Calbindin	Mouse	1∶300	Sigma-Aldrich, St. Louis, MO	C9848 (clone CB955)
G_o_α	Mouse	1∶500–1∶1,000	Millipore, Bellerica, MA	MAB3073 (clone 2A)
Glutamic Acid Decarboxylase, 65 kDa (GAD-65)	Mouse	1∶500–1∶1000	Developmental Studies Hybridoma Bank,University of Iowa, Iowa City, IA	GAD-6 (clone GAD-6)
Glutamine synthetase	Mouse	1∶1000	Millipore, Bellerica, MA	MAB302 (clone GS6)
Peanut Agglutinin (PNA)	–	1∶20	Invitrogen-Molecular Probes, Carlsbad, CA	L21409
Protein Kinase C (PKC)	Rabbit	1∶1000–1∶2000	Sigma-Aldrich, St. Louis, MO	P-4334
Wheat germ agglutinin (WGA)	–	1∶50	Invitrogen-Molecular Probes, Carlsbad, CA	W11261

Frozen sections (10–15 µm thick) of eyecups fixed in 4% paraformaldehyde were fluorescently labeled and imaged as described previously [18]. Cell-specific markers included the 65 kDa isoform of glutamic acid decarboxylase (GAD-65), G_o_α, glutamine synthetase, calbindin, and the α isoform of protein kinase C (PKC). Wheat germ agglutinin (WGA) conjugated to AlexaFluor-488 and peanut agglutinin (PNA) conjugated to AlexaFluor-568 were used to assess the rod and cone-specific domains of the interphotoreceptor matrix. Goat anti-mouse and goat anti-rabbit secondary antibodies conjugated to AlexaFluor-488 or -568 (Invitrogen-Molecular Probes, Carlsbad, CA) were used at a dilution of 1∶200 to visualize primary antibody binding.

All antibodies and lectins were diluted in blocking agent consisting of 10% normal goat serum +5% BSA +1% fish gelatin +0.5% triton X-100 in Hank’s buffered saline solution. Images were captured using an Olympus IX70 fluorescence microscope fitted with a QiCAM camera controlled by QCapture software (QImaging, Surrey, British Columbia, Canada). Image scale was set and images were imported into Photoshop software (Adobe Systems, Mountain View, CA) for preparation of figures.

### Electroretinography

ERGs were recorded from the corneal surface of mice using an LKC UTAS-E 3000 Ganzfeld (LKC Technologies, Inc., Gaithersburg, MD) and analyzed with EM for Windows software (LKC Technologies, Inc.) as previously described [16].

### Assessment of Cell Numbers

Image montages derived from semithin resin sections spanning the vertical meridian of the retina and passing through the optic nerve head were captured at 20× magnification using a Zeiss microscope (Carl Zeiss Meditec, Dublin, CA) and AxioVision software (Carl Zeiss Microscopy, Thornwood, NY). Using Adobe Photoshop CS (Adobe, Mountain View, CA), regions of 25.4 µm by 33.9 µm in the outer nuclear layer (ONL) were selected for cell counting at intervals of every 84.7 µm from the optic nerve head to the periphery of the retina. All nuclei fully contained within each area were counted. Cell numbers in the INL were assessed similarly.

### In situ TUNEL Analysis

Eyes collected from P1 pups were fixed in 4% paraformaldehyde for two hours, processed, embedded in paraffin blocks, and cut into 5 µm sections. Apoptosis in the neuroblastic layer (NBL) was determined by TUNEL labeling using the In Situ Cell Death Detection kit (Roche, Indianapolis, IN) with fluorescein as the fluorophore. Labeled sections were mounted in ProLong Gold Antifade Reagent mounting medium containing DAPI (Invitrogen, Carlsbad, CA). For each mouse, the total number of TUNEL-positive nuclei in the entire NBL from three to four different sections was averaged for analysis. Sections from four non-transgenic (wild type, referred to as wt henceforth) and four super p53 mice were analyzed.

### Statistical Analysis

Unless otherwise stated, statistical significance was determined using one-way analysis of variance (ANOVA) with Bonferroni *post hoc* multiple pairwise comparison tests (Prism, GraphPad Software, San Diego, CA). Statistical significance was accepted if *p*<0.05. Data are presented as mean ± SEM.

## Results

### Super p53 Mice Exhibit Reduced Photoreceptor Function

**Figure 1:**
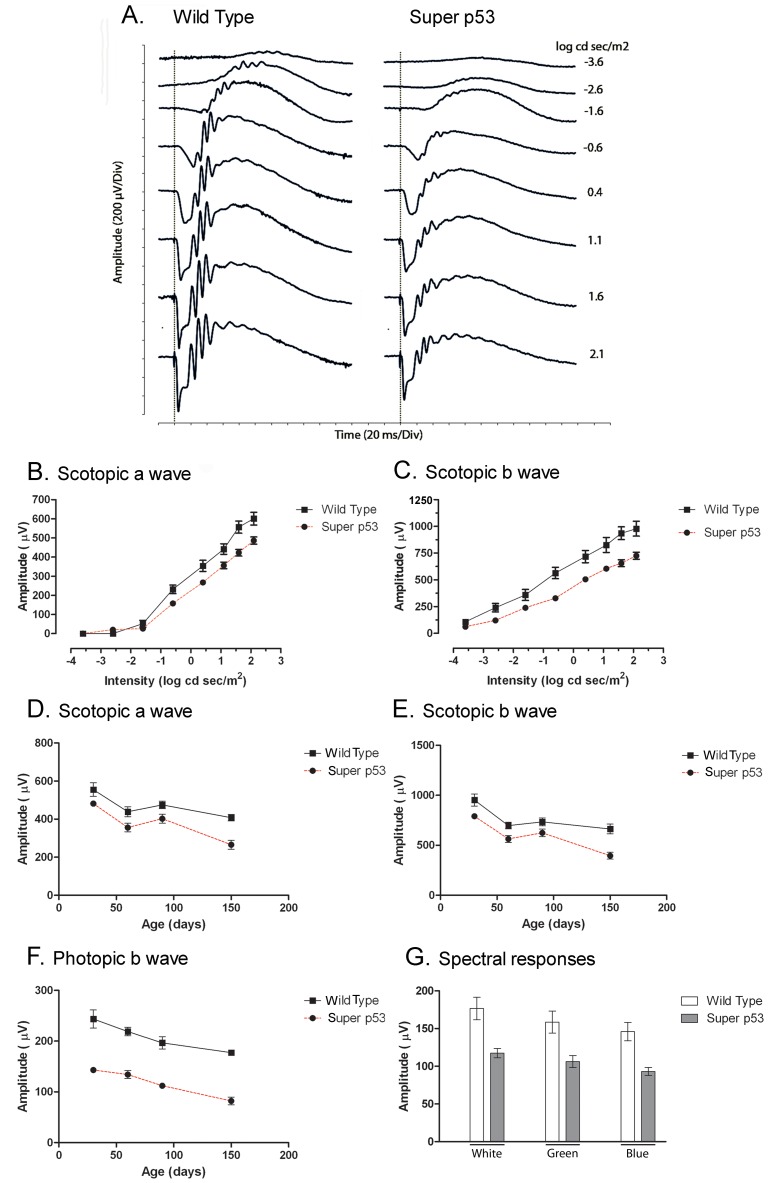
Electroretinographic (ERG) analysis of the super p53 mouse. (**A**) ERG responses from P30 wild type (left) and super p53 (right) mice to a series of stimulus intensities spanning a several log unit range presented to the dark-adapted mouse eye. Dashed vertical lines represent the time of presentation of stimulus. Amplitude of the dark-adapted, scotopic a wave (**B**) and b wave (**C**) plotted as a function of stimulus intensity. (**D**) Amplitude of the scotopic a wave plotted as a function of mouse age. (**E**) Amplitude of the scotopic b wave plotted as a function of mouse age. (**F**) Amplitude of the light-adapted photopic b wave plotted as a function of mouse age. Solid squares represent responses from wild type mice; filled circles represent the super p53 mouse responses. Symbols represent the average obtained from testing 6 to 12 transgenic animals; error bars represent SEM. (G) Spectral electroretinography. Recordings of maximal b wave responses to either a 400 nm flash (blue) or to a 500 nm flash (green) made from P90 wt (filled bars) or super p53 (open bars) mice. Symbols represent the average obtained from testing 6 transgenic animals; error bars represent SEM. The responses marked by “white’ represent the full photopic b wave response.

To determine whether increased steady state levels of p53 affected retinal function, scotopic and photopic ERGs were performed on age-matched super p53 mice and wt controls. Figure 1 presents dark-adapted ERG responses recorded from wt and super p53 mice to flash stimuli spanning a range of intensity of several log units. This range includes light intensities that elicit purely rod-driven (scotopic) responses, mixed rod- and cone-driven responses, and purely cone-driven (photopic) responses (Figure 1A).

The ERG response of wt mice at the lowest light intensity was dominated by the positive-going b wave, which reflects primarily the activity of rod bipolar cells. At increasing intensities, the scotopic b wave was preceded by the negative-going a wave, which represents the collective response of the rods. In super p53 mice, both scotopic a and b wave amplitudes were reduced and mostly fell outside the normal range observed for wt mice at all light intensities (Figure 1B&C). However, there were no changes in implicit time (∼6 milliseconds) of the a wave between super p53 and wt mice (data not shown). Developmentally, the scotopic a and b wave amplitudes of super p53 mice started significantly below the wt amplitudes at P30 and remained lower than wt for all ages examined (Figure 1D&E) but did not show any additional progressive decline over time.

The reduction in ERG response was not limited to rod-driven responses. Cone-driven photopic ERG responses also were reduced in super p53 mice compared to wt controls (Figure 1F). The reduction (∼41%) in photopic b wave amplitude in super p53 mice at P30 was proportionally much larger than the reduction in either the scotopic a wave (∼13%) or the b wave (∼17%) amplitude. To determine whether the reduction in the photopic response from super p53 mice resulted from a selective functional deficit in either green or blue cones, spectral ERGs were performed. The responses elicited from both cone types in post-natal day (P) 90 super p53 mice were reduced (Figure 1G), suggesting that the functional deficit was not cone type-specific. The age-dependent reduction in scotopic and photopic ERG responses from super p53 retinas paralleled that of wt mice, suggesting that the functional deficit initially arose during retinal development and was not followed by any additional progressive functional deficits that were p53-dependent.

**Figure 2:**
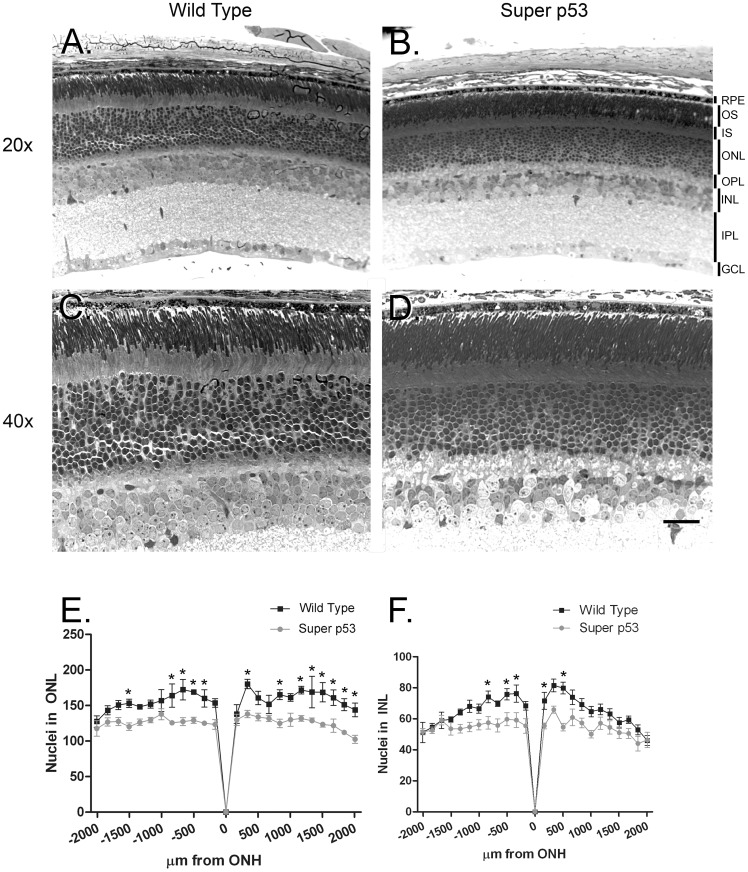
Histologic examination of super p53 retina. Light micrographs of retinal cross-sections taken from Wild Type (**A&C**) and Super p53 (**B&D**) mice. **A&B** present images obtained at 20X and **C&D** present images obtained at 40X. RPE: pigment epithelium; OS: outer segments; IS inner segments; ONL: outer nuclear layer; OPL: outer plexiform layer; INL: inner nuclear layer; IPL: inner plexiform layer; GCL: ganglion cell layer. Bar is equal 50 µm. **E**. The number of nuclei in the ONL was counted along the vertical meridian at each indicated distance from the optic nerve head (ONH, 0) from inferior to superior retinal margin (n7–8 mice). **F**. The number of nuclei in the INL was counted along the vertical meridian at each indicated distance from the optic nerve head (0) from inferior to superior retinal margin (n7–8 mice). P values for points marked by an asterisk ranged between <0.001 and <0.05.

The observed reductions in scotopic and photopic ERG responses from super p53 mice potentially could arise from degenerative changes in photoreceptors and/or higher-order neurons in the inner retina. Alternatively, the reduced ERG responses might result from functional changes unrelated to cell loss. Examination of P30 wt and super p53 retinas at the light microscopy level showed normal retinal lamination of the p53 mouse retina (Figure 2A–D) although the number of cells in the ONL and in the INL were reduced (Figure 2E&F). Electron microscopy showed preservation of normal ultrastructure (Figure S1).

Quantification of the difference in the number of photoreceptor nuclei at P30 in wt and super p53 mice along the vertical meridian of the retina showed that photoreceptors were lost equally across the retina of the super p53 mouse retina (Figure 2E&F). The loss of photoreceptors from the super p53 retina is consistent with the reduced ERG responses in super p53 mice. To determine whether photoreceptor loss in the super p53 retina affected rod and cone photoreceptors differentially, we assessed cone numbers in the retinas of super p53 and age-matched wt control mice by counting cone photoreceptors identified by M- and S-opsin labeling in vertical sections (Figure S2) and in retinal whole mounts (data not shown). Cone numbers in the super p53 and wt retina were similar in both superior and inferior retinal regions. Consistent with these findings, western blot analysis showed no difference in cone opsin levels between the super p53 and wt retinas (data not shown). The rod and cone-specific domains of the interphotoreceptor matrix (IPM), labeled using WGA and PNA, also appeared unaffected in the super p53 retina (data not shown). Together, these data indicate that constitutive overexpression of p53 reduced the total number of photoreceptors but did not cause cone loss. Thus, reduced cell numbers in the ONL of the super p53 retina must reflect a selective reduction in the numbers of rods.

The reduced b wave amplitudes observed in the super p53 mouse ERG, particularly at low light intensities that evoke rod-driven responses, might arise from the reduced number of rods. However, a reduction in the number of inner retinal neurons, particularly bipolar cells, also might contribute to reduced b wave amplitude, particularly for light intensities that drive cone-mediated responses since cone number in the super p53 retina is normal. Therefore, we similarly quantified cell numbers in the INL along the vertical meridian of the retina. These analyses showed that the INL of the super p53 mouse had reduced numbers of cells compared to age-matched wt control mice (Figure 2F). Thus, the reduced numbers of cells in the INL may contribute to the reduced amplitude of the scotopic b wave in the super p53 mouse. Furthermore, the reduced numbers of cells in the INL, rather than defects in cones, may be responsible for the reduced photopic b wave amplitude observed in super p53 mice.

Immunolabeling with markers for specific populations of cells in the INL showed that all cell types examined were present in the super p53 retina and showed morphology comparable to the same cell types in the wt retina (summarized in Table 3). Cell types examined included rod bipolar cells (PKC, shown in Figure S3), ON-bipolar cells (G_o_α), horizontal cells (calbindin), GABAergic amacrine cells (GAD-65), and Müller glial cells (glutamine synthetase) (data not shown). These results suggest that although p53 over-expression reduced the total number of cells in the INL, the cell populations present in the INL were appropriate and showed their normal cell-specific morphological characteristics.

**Table 3:** Normal cell-specific morphology or distribution of retinal cells in the super p53 retina.

Cell- or synapse-specific marker	Wild type	Super p53
*Photoreceptors and IPM*		
PNA	IPM surrounding cone outer segments; flat contacts withOFF-cone bipolar cells at cone terminals	Comparable to wild type
WGA	IPM surrounding rod and cone outer segments	Comparable to wild type
*Bipolar Cells*		
PKC-α	Rod bipolar cell dendrites, cell bodies, axons and theirterminals in the innermost IPL	Comparable to wild type
Goα	All ON-type bipolar cell bodies and dendrites. Other,non-bipolar cell processes in IPL	Comparable to wild type
*Horizontal cells*		
Calbindin	Horizontal cell bodies, dendrites and axons in the OPL	Comparable to wild type
*Amacrine cells*		
GAD-65	GABAergic amacrine cells and their processes in IPL	Comparable to wild type
*Glial cells*		
Glutamate synthetase	Müller cells and astrocytes	Comparable to wild type

These studies showed that overexpression of p53 led to functional deficits and reduced cell numbers in the ONL and INL in the super p53 mouse retina in the absence of further progressive p53-dependent functional decline or cell loss, but did not establish how these defects originated. The deficits observed in the super p53 retina could arise from either increased developmental apoptosis or from altered cell production during retinogenesis.

**Figure 3:**
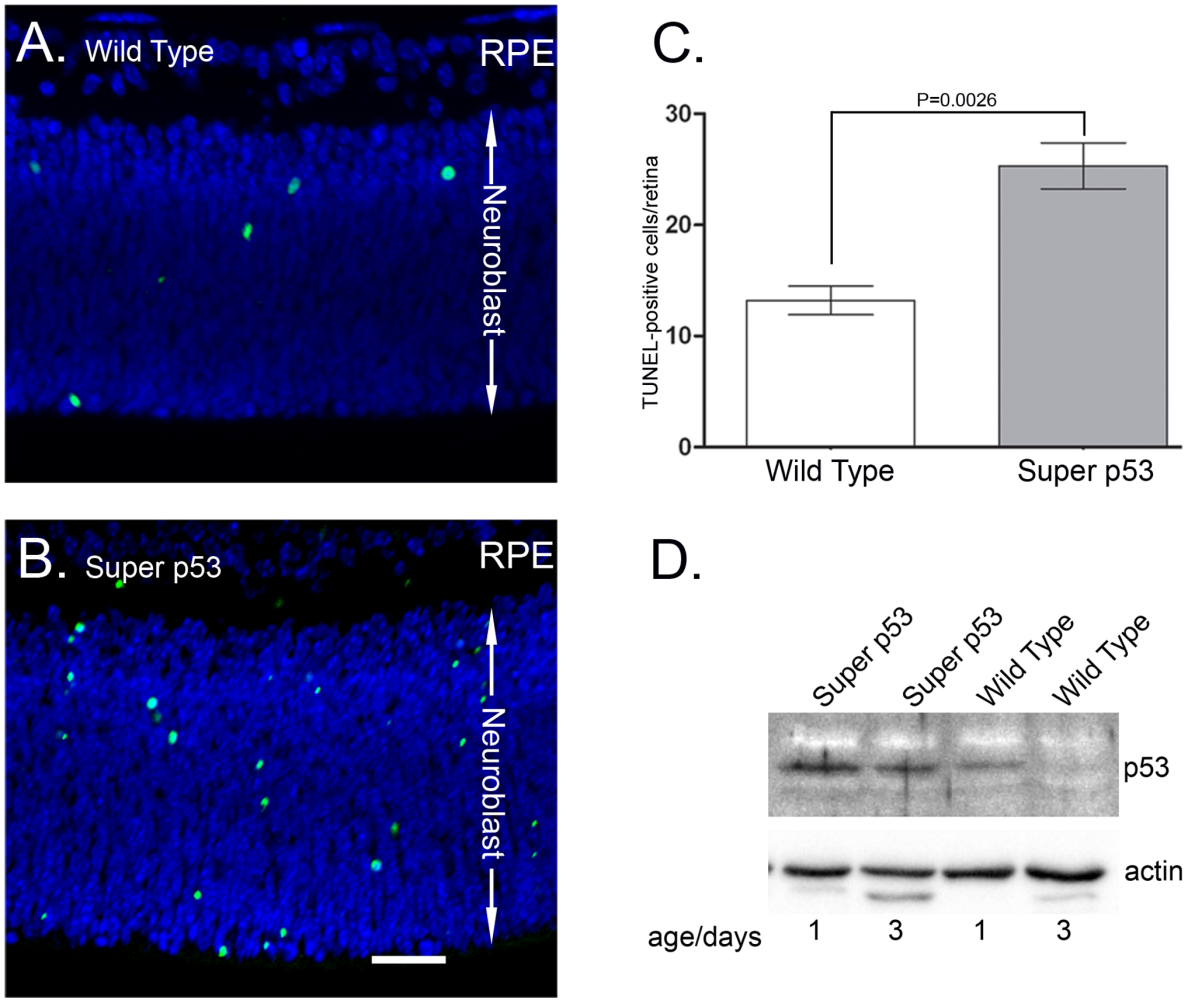
TUNEL labeling of cross sections of P1 retina. TUNEL labeling showed increased apoptotic death in the neuroblastic layer (NBL) of P1 super p53 mice (**B**) when compared to wt retinas (**A**). TUNEL-positive cells are green; nuclei (blue) were stained with DAPI. **C.** Bar graphs showing the increase in the total number of apoptotic cells in a cross section of the retina of the super p53 mouse at P1. N3–4 sections from 4 mice of each genotype. Bars represent SEM. Scale bar50 µm. D. Immunoblot analysis of retinal extracts from super p53 and wild type mice at P1& P3. Upper blot was probed with anti-p53 antibody then stripped and reprobed with anti-actin antibody.

To test whether transgenic p53 expression led to increased developmental apoptosis, TUNEL staining was performed on retinal sections from P1 retinas from both super p53 and wt mice. The transgenic expression of p53 increased numbers of TUNEL-positive cells in the super p53 retina compared to the wt retina (Figure 3). The increase in TUNEL positive cells in the super p53 retina suggests that the super p53 retina produces a normal number of cells but undergoes a higher degree of developmental cell death, ultimately leading to a reduced final number of retinal cells and reduced functional competence. To confirm that the increased apoptosis in the super p53 retina is associated with increased levels of p53, immunoblots were performed on retinal extracts at early retinal developmental stages. As shown in Figure 3D, the levels of p53 are considerably higher in super p53 at both P1 and P3. However, the levels of p53 drop at P3, suggesting that due to the inclusion of its endogenous promoter the transgenic p53 mimics the endogenous p53 in its developmental pattern of expression.

### Effect of HIP Transgenic p53 Expression in Photoreceptors Mimics that Observed in Super p53 Mice

The super p53 mouse clearly exhibited a developmental phenotype. However, because it is a transgenic mouse, it is possible that the transgene may have integrated into a locus in the genome that modulates retinal development. Furthermore, the transgene in the super p53 mouse is regulated by its native promoter, which shows dramatic transcript downregulation by E18 [9], making it difficult to assess the consequences of continued p53 expression in the adult retina.

To address these questions, the HIP construct was generated and injected into E1 mouse embryos. The injection yielded four potential HIP founder mice. All four potential founders successfully passed the transgene to their offspring in a Mendelian fashion. Southern blot analysis revealed that each of the founders contained multiple copies of the transgene integrated into a single site (data not shown) but showed varying levels of transgene expression.

**Figure 4:**
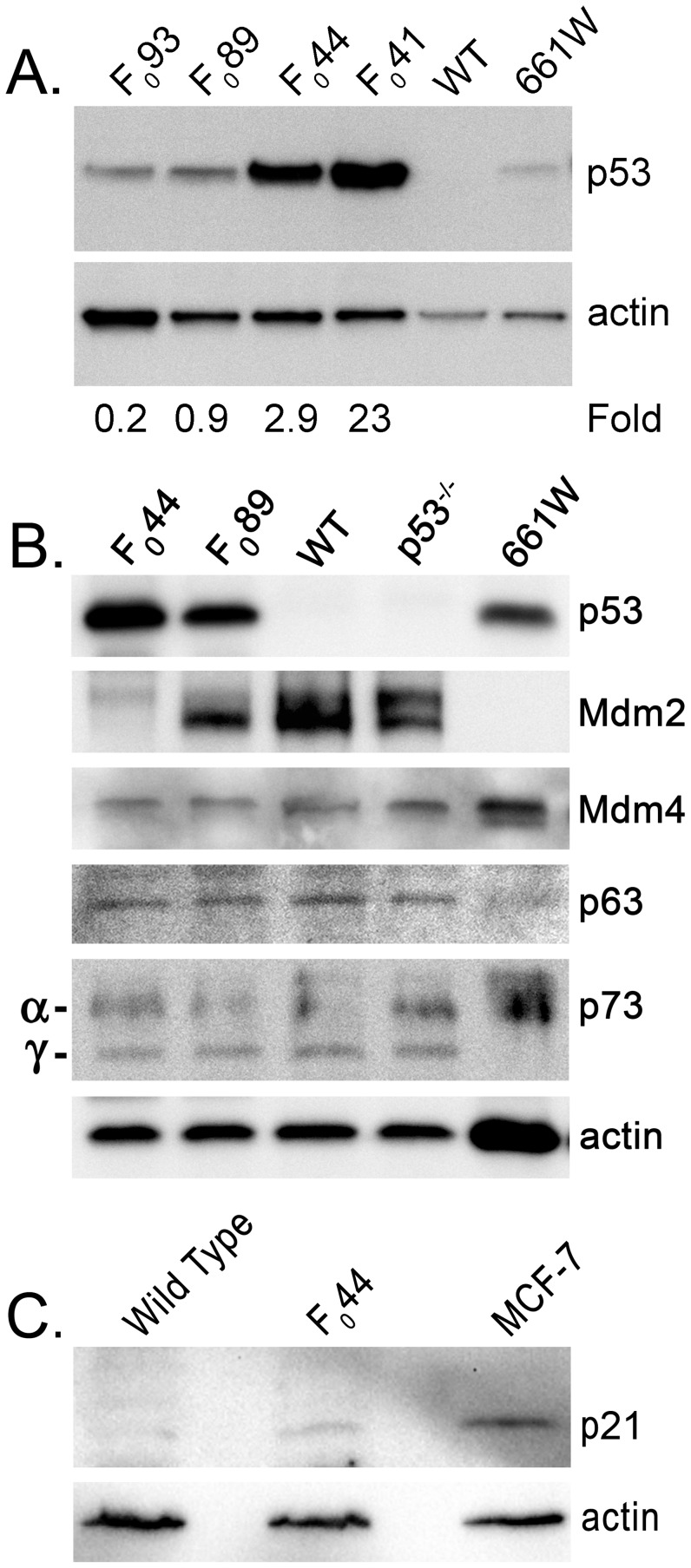
Pattern of expression of p53, its family members, and its regulators in HIP retinas. (**A)** Western blot analysis showing the levels of expression of p53 in four transgenic HIP families relative to its levels in 661W cells, a continuously dividing cone photoreceptor cell line. **(B)** Levels of Mdm2, Mdm4, p63 and p73 were determined in two of the HIP families. Actin served as a loading control**. (C)** Immunoblot demonstrating the increased levels of p21 in HIP retinas at P6–7.

Immunoblot analysis of retinal lysates collected at P30 showed that the HIP mouse lines generated from F_0_93, F_0_89, F_0_44, and F_0_41 expressed increasing amounts of p53 protein (Figure 4A). Compared to 661W cells, a continuously dividing cone photoreceptor cell line [19], founder F_0_93 expressed 0.2× the amount of p53, F_0_89 expressed 0.9×, F_0_44 expressed 2.9×, and F_0_41 expressed 23×. Due to their moderate levels of expression of transgenic p53, the HIP mouse lines generated from F_0_89 and F_0_44 were chosen for subsequent studies.

**Figure 5:**
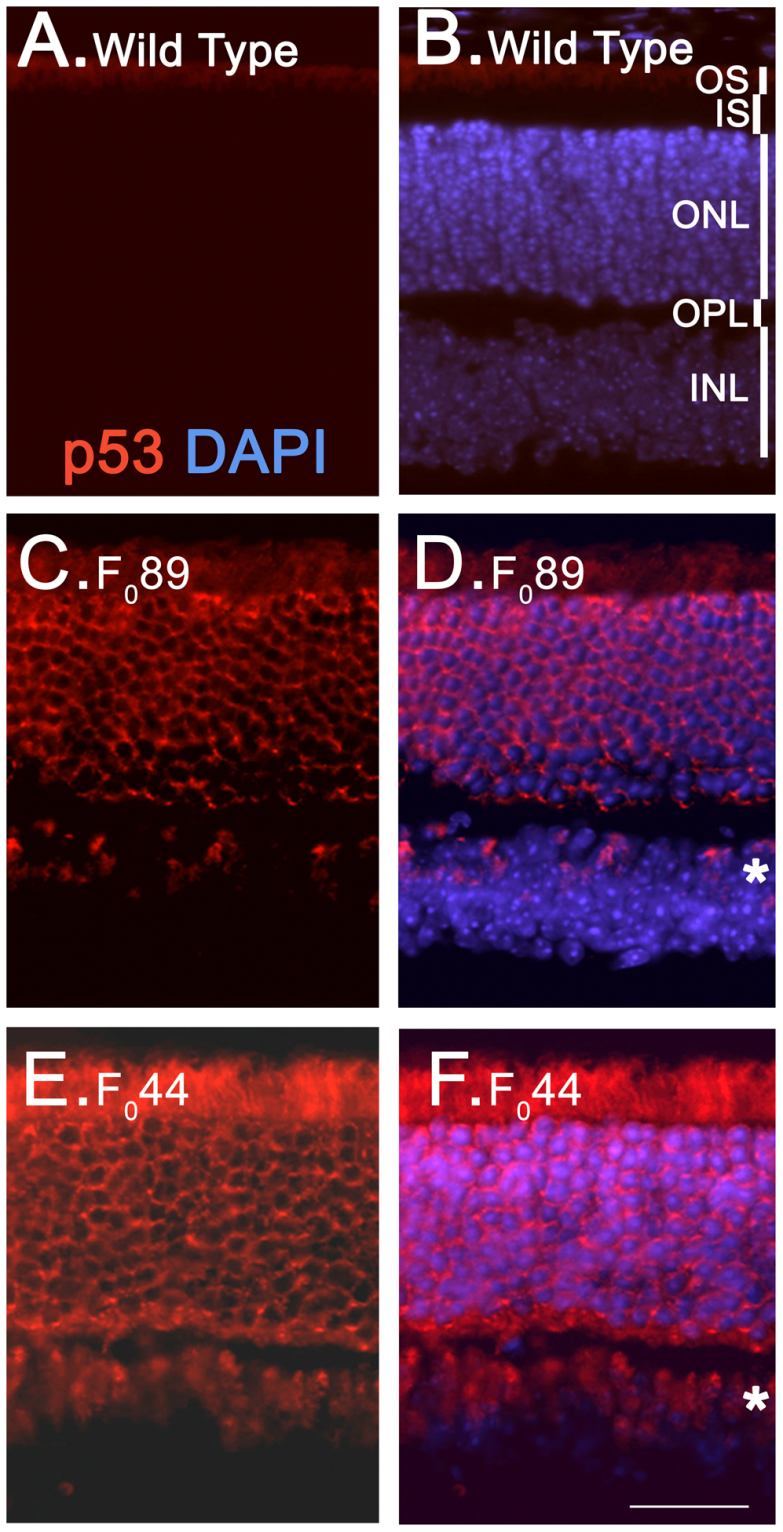
Immunohistochemical localization of p53 in two of the HIP mouse lines. Retinal sections from P30 wild type (**A&B**), F_0_89 (**C&D**) and F_0_44 (**E&F**) mice were labeled using an anti-p53 antibody (red). Asterisks in **D&F** point to p53-expressing cells in the INL of both HIP families. OS outer segments; IS inner segments; ONL outer nuclear layer; OPL, outer plexiform layer; INL, inner nuclear layer. Scale bar20 µm.

To confirm that expression of transgenic p53 driven by the hIRBP promoter was restricted to photoreceptors, IHC was performed on P30 HIP and wt retinal sections. As expected, transgenic p53 was expressed in the retina of both HIP F_0_89 and HIP F_0_44 at levels reflective of that observed on immunoblots (Figures 4 and 5). Transgenic p53 expression was localized to the inner segment (IS), which contains the cytosolic fractions of the rod and cone photoreceptors, and the ONL, which consists of the rod and cone photoreceptor cell bodies.

In addition, p53 signal was also detected in the distal INL near the OPL (asterisks, Figure 5C–F), a region that normally contains horizontal and bipolar cells but not photoreceptors. Because the hIRBP promoter was utilized in the generation of the HIP construct, expression was expected to be limited to the photoreceptors of both rod and cones [20]. During normal retinogenesis, dividing photoreceptor progenitor cells migrate within the NBL [21]. As these progenitors permanently exit the cell cycle, they migrate toward the outer region of the retina where they differentiate into photoreceptors that later establish the ONL. Photoreceptor cells that fail to migrate into the nascent ONL and/or are unsuccessful in establishing proper connections normally undergo apoptosis [22].

In order to determine whether the expression of transgenic p53 in the INL was due to ectopic expression in INL cells or to the mislocalization of p53-overexpressing photoreceptors, the HIP transgenic mice were bred with the *rd* mouse, which has a mutation in the β-subunit of phosphodiesterase that causes all rod photoreceptors to degenerate by P21 [23]–[25], leaving a single row of cone nuclei in the ONL. The retinas of F_0_89 and F_0_44 HIP mice bred onto the *rd/rd* background (HIP^rd/rd^) showed degeneration of all rods in the ONL and the elimination of all p53-expressing cells from the INL (Figure S4). The specific loss of p53 labeling from the INL in the retina of HIP^rd/rd^ mice indicates that the p53 labeling in the INL of HIP mice on the wt background was due to mislocalized photoreceptors rather than ectopic expression of transgenic p53 in horizontal or bipolar cells.

The majority of transgenic p53 was observed in the cytoplasm and the perinuclear region of photoreceptors (Figure 5). Overexpression of cytoplasmic p53 has been observed in some cancers [26]–[33] and during apoptotic signaling [34]. Mouse double minute 2 (Mdm2) or Mdm4, two p53 regulators, can promote p53 nuclear export [35]. If cytoplasmic sequestration of p53 was Mdm2- or Mdm4-dependent, the levels of Mdm2 and/or Mdm4 should have changed. However, Mdm4 did not show altered levels by immunoblot analysis in HIP F_0_44 or HIP F_0_89 retinas (Figure 4B), suggesting that Mdm4 does not play a role in the cytoplasmic sequestration of transgenic p53. We did observe reduced levels of Mdm2 in the HIP F_0_89 retina and little, if any, Mdm2 expression in the HIP F_044_ retina (Figure 4B). This might arise from downregulation of Mdm2 by increased levels of p53 or the integration of the transgene into the Mdm2 locus. However, homozygous HIP F_0_44 and homozygous HIP F_0_89 mice are viable (data not shown) while homozygous Mdm2 knockout mice are not [36], suggesting that Mdm2 downregulation arose from transgenic p53 expression rather than integration of the transgene into the Mdm2 locus. This is further supported by the fact that the dividing 661W cone photoreceptor cell line expressed substantial levels of p53 in the absence of detectable Mdm2 (Figure 4B).

The p53 family of proteins also includes p63 and p73, which are known to play essential roles in development [37]–[42]. However, increased levels of p53 in the retinas of HIP transgenic mice caused no observable changes in levels of either p63 or p73 in comparison to wt (Figure 4B), suggesting that overexpression of p53 did not lead to compensatory changes in the expression of other members of the p53 family.

To determine the effects of increased expression of p53 on its target genes, the levels of p21 were assessed on immunoblots of retinal extracts from P6–7 mice. As shown in Figure 4C, the levels of p21 are increased in HIP retina (∼3X) when compared to their non-transgenic counterparts, indicating that elevated p53 levels do in fact alter the expression of its target genes.

**Figure 6:**
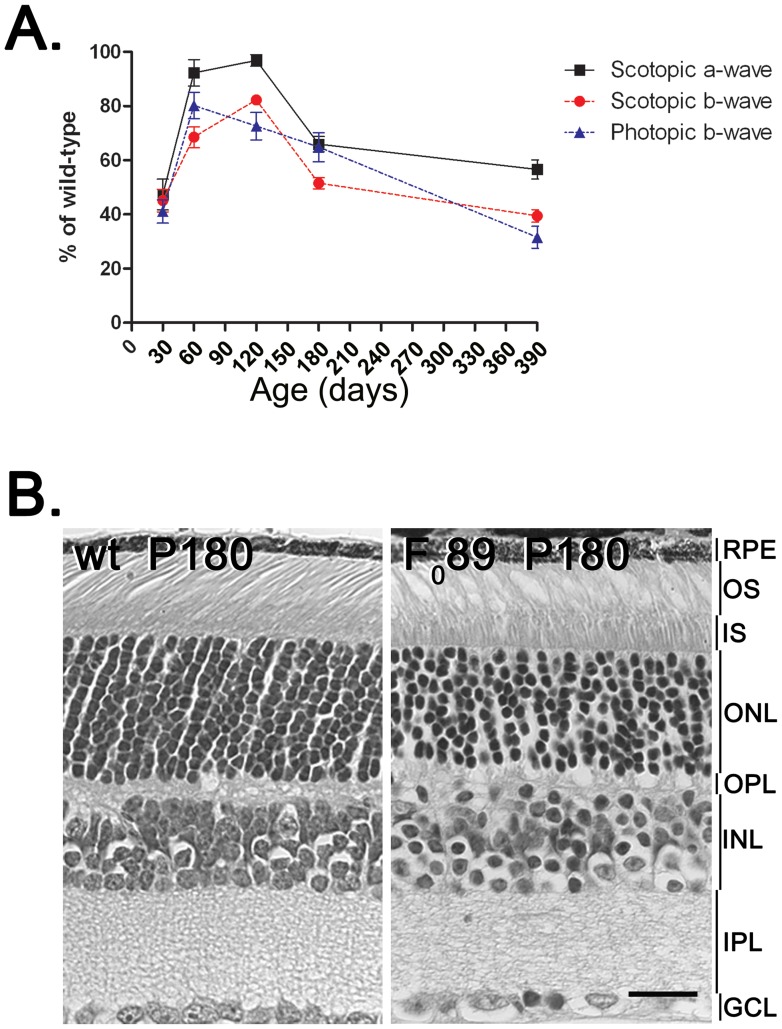
Functional and histologic analyses of the HIP F_0_89 retina. **A.** Developmental electroretinographic analysis of scotopic a and b waves responses and the photopic b wave responses from the HIP F_0_89 retinas presented as percent of wt. **B.** Histologic cross sections of wild type (wt) and HIP F_0_89 retinas. Scale bar50 µm.

To determine whether overexpression of p53 in the retina of HIP mice led to functional deficits similar to those observed in the super p53 mouse, ERGs were performed on the HIP F_0_44 line (Figure 6A). Scotopic ERG recordings show that ERG responses started at lower levels than those recorded from wt, reached almost wt levels between P60 and P120, and then declined as the mice aged. Furthermore, the age-related decreases in the scotopic a and b waves of HIP F_0_44 mice paralleled one another. Cone-driven function, as determined by the photopic b wave, also showed a reduced response at P30 that then improved somewhat by P60, reaching about 80% of wt levels. However, after P60 the photopic b wave then declined steadily with age.

The early reduced responses might arise from either developmental delays or secondary effects on retinal function. The apparently normal retinal structure of the HIP transgenic mice (Figure 6B) suggests that secondary functional effects may be a more likely cause than developmental delay. However, histologic examination of retinal sections from HIP mice revealed degenerative changes, evidenced by a reduced number of nuclei in the ONL (Figure 6B), suggesting that the progressive decline in ERG responses is likely to reflect degenerative changes.

## Discussion

To determine the functional role of p53 in the retina, the super p53 mouse, a line that globally overexpresses p53 under the control of the endogenous p53 promoter, and transgenic HIP mouse lines, which overexpress p53 specifically in photoreceptor cells under the control of the hIRBP promoter, were studied. The studies performed in the super p53 mouse indicate that developmental overexpression of p53 in the retina leads to the selective loss of rod photoreceptors, but leaves the cone photoreceptor population apparently intact. TUNEL labeling studies indicate that the reduced rod population in the super p53 retina arises from increased apoptotic rod cell death rather than from failure of rod generation. The selective loss of rods also is consistent with the functional deficits shown by the diminished a and b waves of the rod-driven ERG in the super p53 mouse. The reduced numbers of cells in the INL of the super p53 mouse also might contribute to the reduced scotopic b wave amplitude, which reflects the activity of cells in the inner retina, particularly rod bipolar cells. The respective contributions of the reduced rod populations and the reduced number of cells in the INL to the decline in scotopic b wave decline are not clear at present.

One possibility for the selective effects of p53 on rods is the fact that p53 protein expression peaks at E18 [9], after most, if not all, cones have already differentiated [43]. In addition, the lack of any further progressive degeneration in the super p53 retina suggests that the transgenic p53 gene is regulated in a manner similar to that of the endogenous p53 gene and that the introduction of the extra copy only leads to increased p53 levels without affecting the onset of expression or the timing of downregulation. This also supports the notion of the absence of co-regulation between p53 allelic genes.

The developmental overexpression of p53 also compromised cone function, as shown by the reduced amplitude of the photopic b wave, even though the cone population of the super p53 mouse retina appeared to be intact. The reduced photopic b wave in the super p53 mouse suggests that the loss of cells from the INL compromises cone-driven retina function, although secondary effects related to the reduced numbers of rods in the super p53 mouse retina also could indirectly contribute to the compromised cone-driven responses.

Developmental overexpression of p53 led to a decrease in the numbers of cells residing in the INL, but precisely how this decrease in cell number occurs is not currently clear. One possibility is that developmental overexpression of p53 might cause a small, generalized increase in cell death across multiple types of inner retinal cells. It is also conceivable that developmental overexpression of p53 might selectively reduce some specific subset of retinal neurons. Although the IHC studies presented here were not exhaustive, it is clear that developmental overexpression of p53 did not compromise the ability of the retina to generate the different classes of inner retina cells, as all classes were present. Furthermore, all specific subtypes of inner retinal cells that were examined showed their normal cell-specific morphological characteristics. Together, these studies suggest that p53 is not critical to the generation and differentiation of the various retinal cell types, but rather is more likely involved in regulating developmental apoptosis and determining the final number of cells in the mature retina.

Results from the HIP studies show that continued expression of p53 into adulthood is deleterious and seems to equally affect both rods and cones, leading to a degenerative phenotype.

Two pieces of information can be gleaned from the above studies: The levels of p53 are tightly regulated, and the timely downregulation of p53 during retinal development is necessary to maintain a healthy number of cells in the retina. Any perturbation in p53 levels can lead to disastrous consequences for the retina.

The continued expression of p53 in HIP retinas beyond E18 caused a delay in the development of retinal function, emphasizing the significance of the downregulation of p53 during retinal development. Alternatively, the developmental functional delay may result from the early expression of p53 in the HIP retina because the onset of expression by the human IRBP promoter is between E10–13 [44].

A surprising finding in the HIP mice is lack of co-regulation of Mdm2 as a result of expression of higher levels of p53. This may reflect a behavior limited to the retina since it has been shown that Mdm2 pattern of expression does not follow that of p53 during early ocular development while that of Mdm4 seems to mimic the pattern of expression of p53 [9]. This is supported by the presence of only Mdm4 in the 661W cells.

Another interesting observation in HIP retinas is the presence of the majority of p53 perinuclearly and in the cytoplasm rather than the anticipated nuclear localization. This may be due to the higher levels of p53 expressed and the relatively unchanged levels of its regulators, Mdm2 and As a result under these conditions p53 may end up interacting with novel binding partners or organelles, altering its signaling and leading to cell death.

Because p53 null mice showed no ocular phenotype in the C57BL×CBA and 129/Sv×C57BL/6 backgrounds, investigators concluded that p53 lacks any biological role in retinal development and homeostasis [6], [45]. Additionally, it has been shown that cell death in the *N*-methyl-*N*-nitrosourea-induced model of retinitis pigmentosa is p53-independent because the pattern of photoreceptor degeneration was similar whether on a wt or p53 null background [39]. In both of these cases, the patterns of expression of the p53 family members p63 and 73 were not examined to determine whether these other members of the p53 family may have compensated for the loss of p53. Both p63 and 73 can regulate the cell cycle and apoptotic cell death in a fashion similar to that of p53 [46], [47]. Although the conclusions made were correct that in certain cases retinal degeneration is p53-independent, it is yet to be determined definitively whether it also independent of all members of the p53 family.

In summary, this report demonstrates that p53 has a major role in developmental apoptosis in the retina and may play an as yet unidentified role in human blinding diseases associated with increased levels of p53 resulting from mutations in the p53 gene.

## Supporting Information

Figure S1 Expression of p53 does not disrupt general retinal ultrastructure. Montages show cross sections that span the entire retina of both Wild Type and super p53 mice. All retinal layers are preserved and the subcellular organization is intact. Scale bar2 µm.
(TIF)

Figure S2 Cone numbers in wild type and super p53 retinas. Whole eyes were sectioned along the superior-inferior axis. All OS structures labeled by IHC using M-opsin or S-opsin-specific antibodies within the first 324.6 µm on either side of the optic nerve head were counted. The graph shows the number of cones per 100 µm. N2 sections from 4–5 mice of each genotype. Bars represent SEM.
(TIF)

Figure S3 Expression of p53 does not specifically alter retinal cell morphology or distribution. Rod bipolar cells immunolabeled for PKC shown. Rod bipolar cells in the wild type (**A**) and super p53 expressing retina (**B**) show comparable morphology and distribution, similar to other cell types tested (see text for details). ONL outer nuclear layer; OPL, outer plexiform layer; INL, inner nuclear layer; IPL, inner plexiform layer; GCL, ganglion cell layer. Scale bars20 µm.
(TIF)

Figure S4 Cross sectional analysis of retinas of wt and super p53 in the *rd/rd* background. Sections were immunolabeled for p53 (red) and rhodopsin (green). Nuclei (blue) are stained with DAPI. Mice from F_0_89 (**A**) and F_0_44 (**C**) were bred to *rd/rd* mice and then backcrossed to generate *rd/rd* mice expressing the p53 transgene from F_0_89 (**B**) and F_0_44 (**D**). Retinal sections from wt (**E**), *rd/rd* (**F**) and p53^−/−^ (**G**) mice served as controls.
(TIF)
