# Supplementary figures and images for: p53 Selectively Regulates Developmental Apoptosis of Rod Photoreceptors
Source: PLoS One. 2013 Jun 20;8(6):e67381. doi: 10.1371/journal.pone.0067381 (PMC3688626; doi:10.1371/journal.pone.0067381)

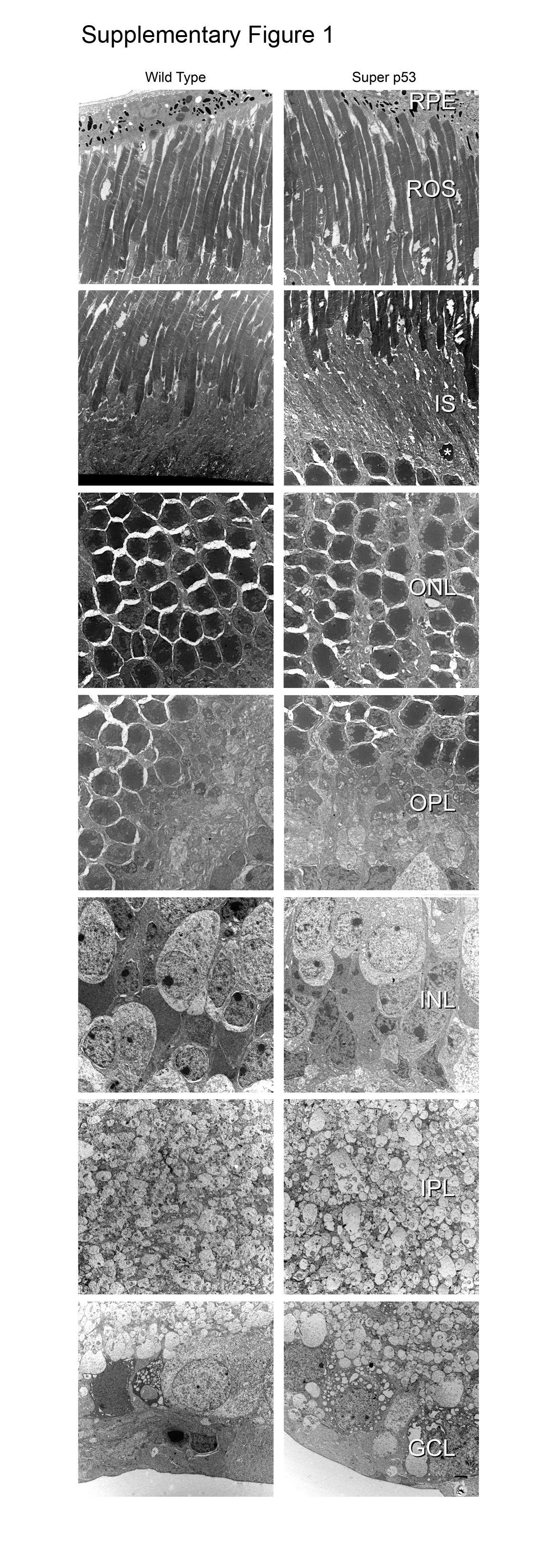

Supplement: Figure S1 — Expression of p53 does not disrupt general retinal ultrastructure. Montages show cross sections that span the entire retina of both Wild Type and super p53 mice. All retinal layers are preserved and the subcellular organization is intact. Scale bar2 µm. (TIF) [file pone.0067381.s001.tif]

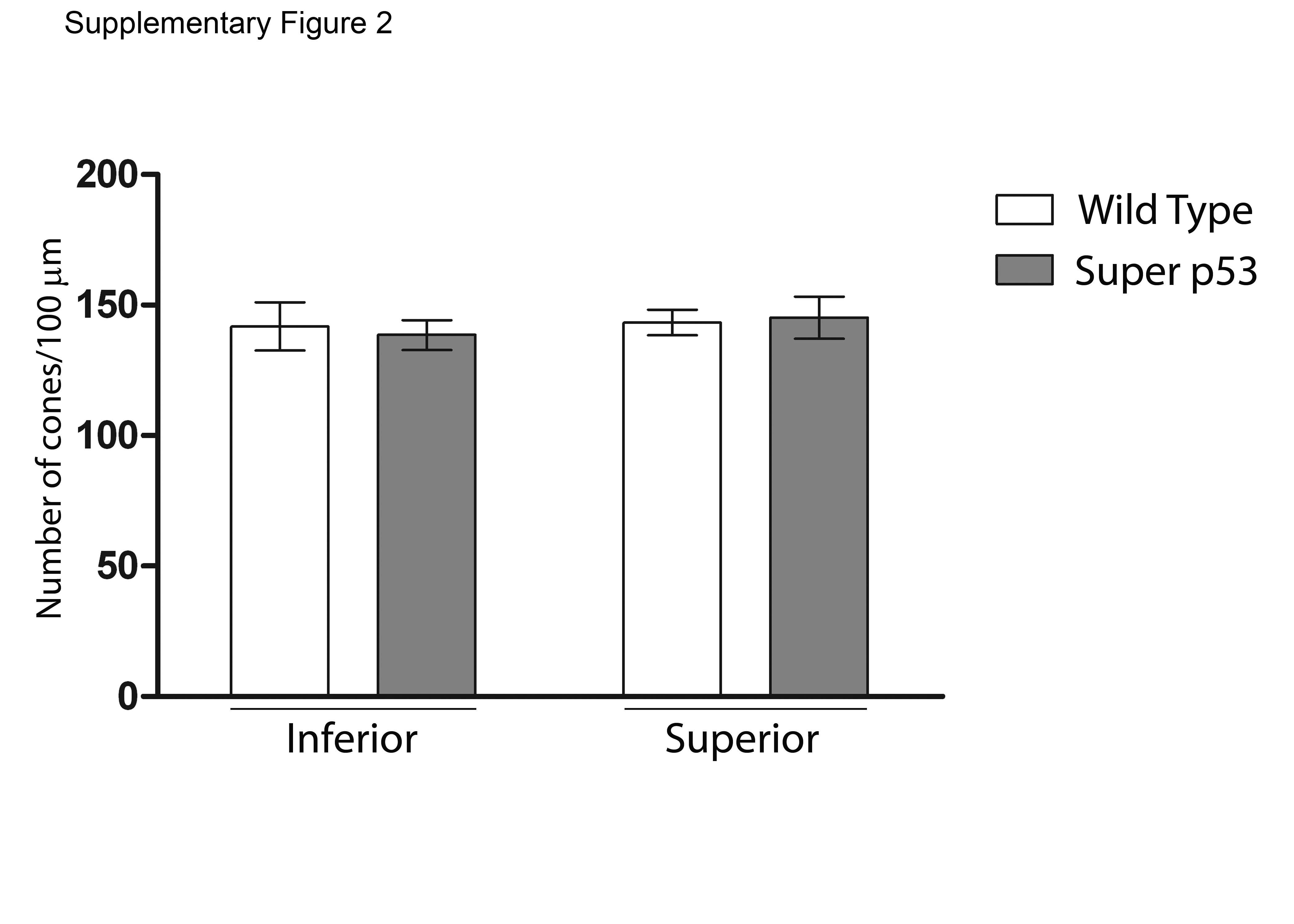

Supplement: Figure S2 — Cone numbers in wild type and super p53 retinas. Whole eyes were sectioned along the superior-inferior axis. All OS structures labeled by IHC using M-opsin or S-opsin-specific antibodies within the first 324.6 µm on either side of the optic nerve head were counted. The graph shows the number of cones per 100 µm. N2 sections from 4–5 mice of each genotype. Bars represent SEM. (TIF) [file pone.0067381.s002.tif]

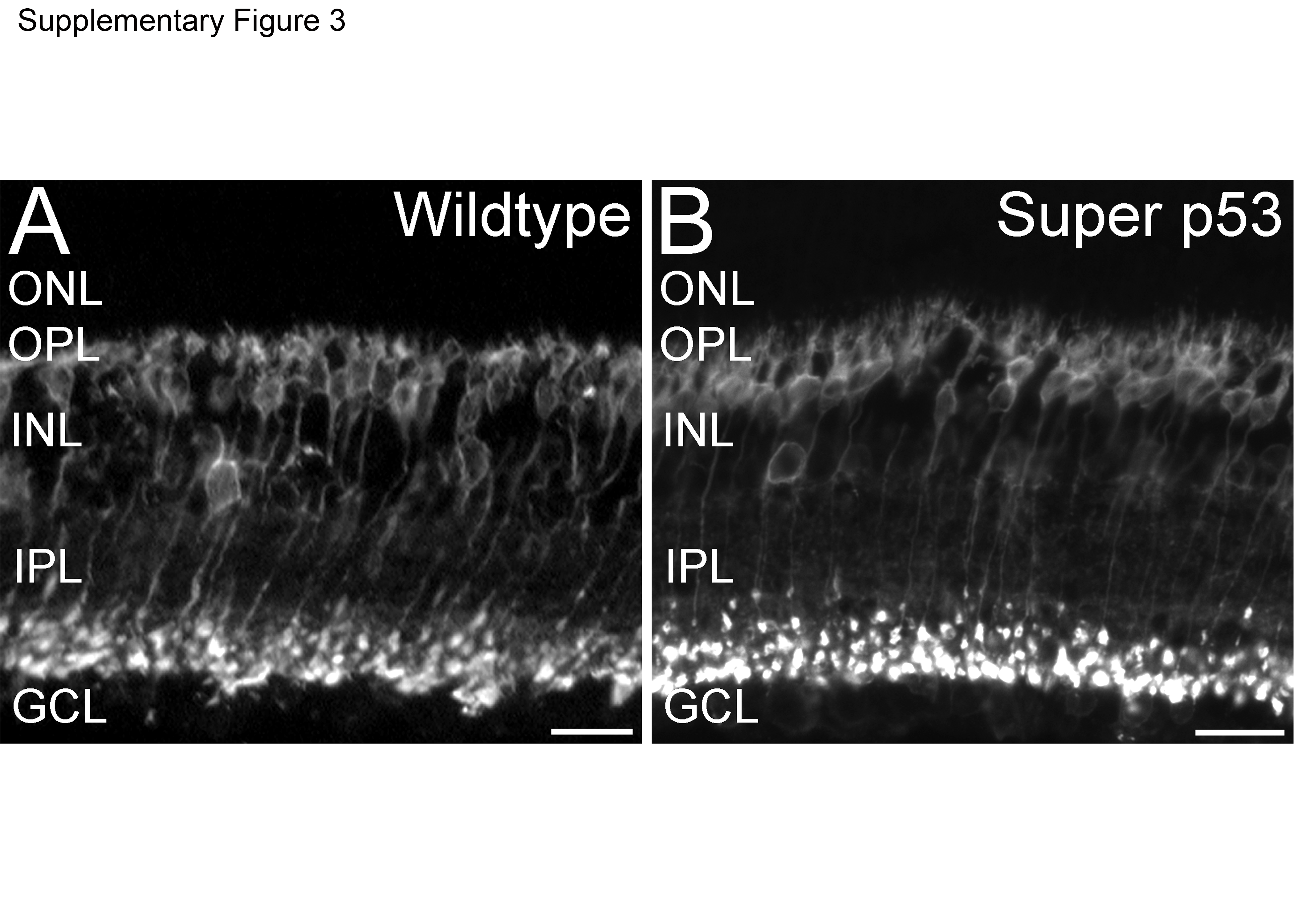

Supplement: Figure S3 — Expression of p53 does not specifically alter retinal cell morphology or distribution. Rod bipolar cells immunolabeled for PKC shown. Rod bipolar cells in the wild type (A) and super p53 expressing retina (B) show comparable morphology and distribution, similar to other cell types tested (see text for details). ONL outer nuclear layer; OPL, outer plexiform layer; INL, inner nuclear layer; IPL, inner plexiform layer; GCL, ganglion cell layer. Scale bars20 µm. (TIF) [file pone.0067381.s003.tif]

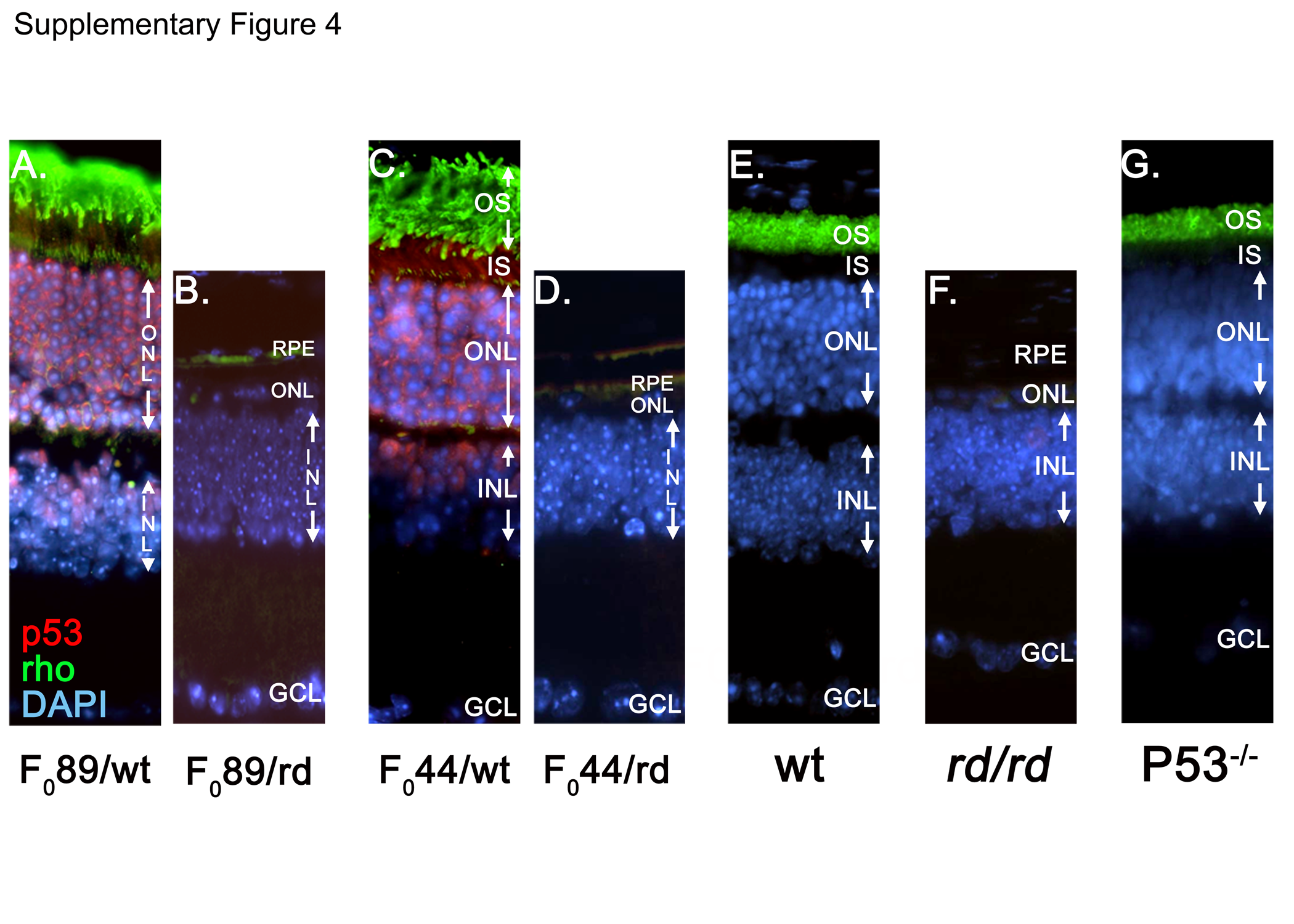

Supplement: Figure S4 — Cross sectional analysis of retinas of wt and super p53 in the rd/rd background. Sections were immunolabeled for p53 (red) and rhodopsin (green). Nuclei (blue) are stained with DAPI. Mice from F089 (A) and F044 (C) were bred to rd/rd mice and then backcrossed to generate rd/rd mice expressing the p53 transgene from F089 (B) and F044 (D). Retinal sections from wt (E), rd/rd (F) and p53−/− (G) mice served as controls. (TIF) [file pone.0067381.s004.tif]
